# Surface Modification of ZrO_2_ Nanoparticles with TEOS to Prepare Transparent ZrO_2_@SiO_2_-PDMS Nanocomposite Films with Adjustable Refractive Indices

**DOI:** 10.3390/nano12142328

**Published:** 2022-07-06

**Authors:** Hanjun Cho, Deunchan Lee, Suyeon Hong, Heegyeong Kim, Kwanghyeon Jo, Changwook Kim, Ilsun Yoon

**Affiliations:** 1 Department of Chemistry, Chungnam National University, Daejeon 34134, Korea; jhjnamoo@naver.com (H.C.); hjdc1278@gmail.com (D.L.); rieh10@cnu.ac.kr (S.H.); hgk205@daum.net (H.K.); jokh7707@naver.com (K.J.); 2 School of Electric Engineering, Kookmin University, Seoul 02707, Korea

**Keywords:** adjustable refractive index, nanocomposite, zirconia nanoparticle, sol–gel method, surface modification

## Abstract

Here, highly transparent nanocomposite films with an adjustable refractive index were fabricated through stable dispersion of ZrO_2_ (*n* = 2.16) nanoparticles (NPs) subjected to surface modification with SiO_2_ (*n* = 1.46) in polydimethylsiloxane (PDMS) (*n* = 1.42) using the Stöber method. ZrO_2_ NPs (13.7 nm) were synthesized using conventional hydrothermal synthesis, and their surface modification with SiO_2_ (ZrO_2_@SiO_2_ NPs) was controlled by varying the reaction time (3–54 h). The surface modification of the NPs was characterized using Fourier-transform infrared spectroscopy, dynamic light scattering, X-ray photoelectron spectroscopy, scanning electron microscopy, transmission electron microscopy, and ellipsometry. The surface modification was monitored, and the effective layer thickness of SiO_2_ varied from 0.1 nm to 4.2 nm. The effective refractive index of the ZrO_2_@SiO_2_ NPs at λ = 633 nm was gradually reduced from 2.16 to 1.63. The 100 nm nanocomposite film was prepared by spin-coating the dispersion of ZrO_2_@SiO_2_ NPs in PDMS on the coverslip. The nanocomposite film prepared using ZrO_2_@SiO_2_ NPs with a reaction time of 18 h (ZrO_2_@SiO_2_-18h-PDMS) exhibited excellent optical transparency (T_average_ = 91.1%), close to the transparency of the coverslip (T_average_ = 91.4%) in the visible range, and an adjustable refractive index (*n* = 1.42–1.60) as the NP content in the film increased from 0 to 50.0 wt%.

## 1. Introduction

Polymers are widely used in our daily lives and in industry because of their easy processing, flexible functions, and diverse applications [1]. Several chemical synthesis methods use modified polymer materials for multiple functionalities while maintaining their basic properties [2]. However, the practical development of polymers has limitations, such as high cost and difficult production [3,4]. Incorporating inorganic nanoparticles (NPs) into polymer matrices allows the polymers to take on the various functions of the added NPs and enhances their mechanical, thermal, optical, and plasmonic properties. Owing to these advantages, nanocomposites have been developed in various research and industrial fields for practical use as high-performance functional materials [1,5,6,7,8,9,10,11,12].

A representative application of nanocomposites is as transparent high-refractive-index materials that can effectively manipulate light. Therefore, these materials can play an essential role in the development of compact lenses, optical fibers, and high-efficiency mobile displays by improving light refraction and extraction [10,11,12]. To realize a transparent nanocomposite with a high refractive index, the following criteria must be satisfied [1]. First, considering applications utilizing the visible region, NPs and polymers should not absorb light in the visible region (400–800 nm). Second, NPs added to increase the refractive index of a nanocomposite should have a high refractive index. Refractive index matching between the NP and polymer must also be considered; therefore, selecting appropriate materials for the NP and polymer is essential [13]. Third, the size of NPs should be negligibly small compared to the wavelength of light to prevent an increase in haze due to light scattering and a reduction in light transmission [13,14]. The agglomerate formation in NPs can cause strong light scattering [14]. Several studies have reported methods for the strengthening of chemical affinity or for the conforming of stable chemical bonds between NPs and polymers to avoid agglomeration [15,16,17,18,19,20,21,22,23,24,25,26,27,28,29].

Due to their high refractive index and transparency in the visible region, ZrO_2_ NPs have been used in the development of transparent nanocomposite films with high refractive indices [25,26,27,28,29]. Many studies investigating the surface modification of ZrO_2_ with organic molecules or polymers in order to avoid ZrO_2_ agglomeration and achieve stable dispersion in the polymer matrix have been published [21,22,23,24,25,26,27,28,29]. A typical example of such materials is a commercial product from Pixelligent Technologies LLC (Baltimore, MD, USA) that uses ZrO_2_ NPs 5–20 nm in size functionalized with organic molecules. However, this product can be used with epoxy, acrylic resins, or specific polymers [30,31,32]. In addition to the surface modification of ZrO_2_ with organic materials, Vossmeyer et al. and Xu et al. reported the surface modification of ZrO_2_ with inorganic materials, such as SiO_2_. However, they did not show the fabrication of transparent nanocomposite films [21,22]. To the best of our knowledge, there have been no reports on the fabrication of transparent nanocomposite film with adjustable refractive indices utilizing ZrO_2_ NPs surface-modified with only a SiO_2_ layer.

**Figure 1:**
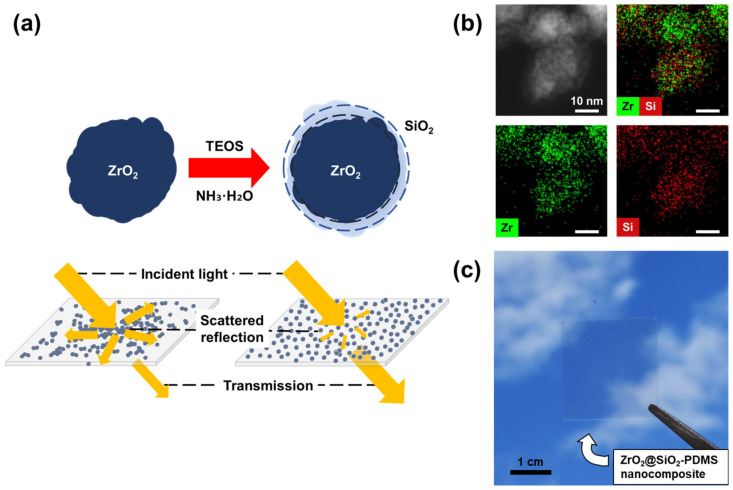
(**a**) Schematic illustration of surface modification of ZrO_2_ NPs using TEOS to prepare a transparent nanocomposite film with adjustable refractive index. (**b**) TEM-EDX images of ZrO_2_@SiO_2_ NPs indicating that the surface of ZrO_2_ NPs was modified with the SiO_2_ layer. (**c**) Photo of ZrO_2_@SiO_2_-PDMS nanocomposite film (100 nm thickness) containing 50 wt% ZrO_2_@SiO_2_ NPs.

For uniform dispersion of ZrO_2_ NPs in a non-polar polymer with high molecular weight, like polydimethylsiloxane (PDMS), modifying the polar surface of NPs with appropriate organic molecules or polymers containing significantly different polarities is difficult [29,30,31,32,33]. Therefore, research on chemical synthesis methods for surface modification of NPs that can be easily accessed at the lab scale is required. In this study, we prepared ZrO_2_ NPs with a size of 13.7 nm through conventional hydrothermal synthesis [22]. As shown in Figure 1, surface modification of ZrO_2_ NPs with SiO_2_ (ZrO_2_@SiO_2_ NPs) was performed through hydrolysis and polymerization of tetraethyl orthosilicate (TEOS) using the Stöber method [22]. We demonstrate that a transparent, thin nanocomposite film with an adjustable refractive index can be fabricated by dispersing ZrO_2_@SiO_2_ NPs in PDMS. The surface modification was characterized by transmission electron microscopy (TEM) with energy-dispersive X-ray spectroscopy (EDX) and X-ray photoelectron spectroscopy (XPS). Fourier-transform infrared spectroscopy (FT-IR) and dynamic light scattering (DLS) confirmed the growth of the SiO_2_ layer on ZrO_2_ NPs, which was controlled by the reaction time of the reaction between the NPs and TEOS (≤54 h). Based on the growth of the SiO_2_ layer, the effective refractive index of the ZrO_2_@SiO_2_ NPs was determined from the aqueous NP solution by ellipsometry. The effective thickness of the SiO_2_ layer (0–4.2 nm) formed on the surface of the ZrO_2_ NPs was calculated using the effective refractive index [34]. After 18 h of reacting with TEOS, the ZrO_2_@SiO_2_ NPs had an appropriate thickness (1.3 nm) for the SiO_2_ layer that allowed stable dispersal in the PDMS matrix with a high refractive index. Depending on the ZrO_2_@SiO_2_ NP content (ZrO_2_@SiO_2_-18h), the refractive index of the nanocomposite films was adjusted from 1.42 to 1.60 in the visible region. The nanocomposite film exhibited excellent optical transparency (T_average_ = 91.1%), close to the transparency of the coverslip (T_average_ = 91.4%).

## 2. Materials and Methods

### 2.1. Materials

Zirconyl chloride octahydrate (ZrOCl_2_·8H_2_O, 98%), urea (CH_4_N_2_O, 99.0–100.5%), citric acid (C_6_H_8_O_7_, ≥99.5%), TEOS (Si(OC_2_H_5_)_4_, 98%), and aqueous ammonia (28 wt%) were purchased from Sigma Aldrich Co. (St. Louis, MI, USA). Ethanol, methanol, 2-propanol, and n-hexane were purchased from Samchun Chemicals Co. (Seoul, Korea). Commercial deionized water (18.25 MΩ, 25 °C) was purchased from Joylife Co. (Gimhae, Korea). A curing agent and PDMS base resin (Sylgard 184) were purchased from Dow Corning Co. (Midland, TX, USA). All the chemicals were used without further purification.

### 2.2. Synthesis of ZrO_2_ NPs

ZrO_2_ NPs were synthesized through conventional hydrothermal synthesis, and the experimental details were modified for our environment [22]. A total of 0.8 g of zirconyl chloride octahydrate, 1.2 g of urea, and 0.4 g of citric acid were added to 25 g of deionized water. The solution was vigorously mixed for 30 min and then transferred to a precleaned Teflon hydrothermal reactor. The reactor was placed into a hydrothermal furnace and heated to 150 °C for 2 h. The reaction proceeded for 12 h, and then the hydrothermal reactor was cooled to room temperature. After cooling, the solution was centrifuged using methanol. Methanol was used for the primary centrifuge because it has greater solubility in urea than ethanol [35]. An additional centrifugation was performed with ethanol. The NPs were dried in a vacuum desiccator and used for TEOS surface modification.

### 2.3. Surface Modification of ZrO_2_ NPs with SiO_2_

Surface modification of ZrO_2_ NPs was performed using the Stöber method, and the experimental details were modified for our environment [22]. A total of 46 mg of ZrO_2_ NPs was dispersed in 1 g of deionized water. The ZrO_2_ NP dispersion and 0.5 mL of aqueous ammonia were added to 20 mL of 2-propanol. The solution was stirred at 1000 rpm at 30 °C, and then 24 μL of TEOS was added to the solution. To control the thickness of the SiO_2_ layer, the reactions were performed at different reaction times of 3, 9, 18, 27, and 54 h. The solution was then centrifuged using 2-propanol, and additional centrifuges were performed with ethanol. The synthesized ZrO_2_@SiO_2_ NPs were then dried in a vacuum desiccator.

### 2.4. Preparation of ZrO_2_@SiO_2_-PDMS Nanocomposites

An equal amount of PDMS mixture and ZrO_2_@SiO_2_ NPs were added to hexane in a mass ratio of 1:1:100 (PDMS mixture:ZrO_2_@SiO_2_ NPs:hexane). The PDMS mixture was prepared by mixing the PDMS base with a curing agent at a weight ratio of 10:1 (PDMS base:curing agent). This mixture was fully dispersed by ultrasonication and dropped on the precleaned substrates (silicon wafer, coverslip). Spin-coating was performed at 1500 rpm for 30 s to produce the 100 nm nanocomposite film. Finally, the ZrO_2_@SiO_2_-PDMS nanocomposite film was cured in a convection oven for 5 h at 80 °C.

### 2.5. Characterizations

The morphologies of the ZrO_2_ and ZrO_2_@SiO_2_ NPs were investigated with TEM (JEOL, JEM-ARM200F, Tokyo, Japan). For qualitative elemental analysis, EDX (Bruker, Quantax 400, Billerica, MA, USA) was performed using TEM. X-ray diffraction (XRD; Bruker, D8 Advance, Billerica, MA, USA) was used to determine the crystal structure of the ZrO_2_ NPs. For the scanning electron microscopy (SEM, Tescan, Clara, Brno, Czech Republic) analysis, the ZrO_2_ and ZrO_2_@SiO_2_ NPs were diluted to 1 g/L in ethanol and drop-casted on silicon wafers. Nanocomposite films were prepared on silicon wafers. The NPs and nanocomposite films were sputtered with Pt prior to SEM analysis. The elemental analysis of ZrO_2_ and ZrO_2_@SiO_2_ NPs was verified using XPS (Thermo Scientific, K-alpha+, Waltham, MA, USA). FT-IR (Shimadzu, IRSpirit, Kyoto, Japan) at the CNU Chemistry Core Facility (Daejeon, Korea) was used to analyze the chemical bonds of the ZrO_2_ and ZrO_2_@SiO_2_ NPs. NPs were diluted to 1 g/L in deionized water and ultrasonicated, and their hydrodynamic diameter was measured using DLS (Malvern Instruments, Zetasizer Nano ZS, Malvern, UK). An ultraviolet-visible spectrophotometer (UV-vis; Agilent Technologies, Agilent 8453, Santa Clara, CA, USA) at the CNU Chemistry Core Facility (Daejeon, Korea) was used to determine the optical characteristics of the aqueous solutions of the ZrO_2_ and ZrO_2_@SiO_2_ NPs. The refractive indices of the NPs and nanocomposite films were measured using an ellipsometer (J.A. Woollam Co., RC-2, Lincoln, NE, USA). NPs were dispersed in deionized water to avoid light scattering and nanocomposite films were prepared on silicon wafers [36]. A lab-built UV-vis spectrophotometer with an integrating sphere was used for the optical characterization of nanocomposite films, and their reflectance and transmittance were measured on a coverslip. The extinction spectrum was obtained from the following relationship:

(1) $$E\left(\lambda \right)+T\left(\lambda \right)+R\left(\lambda \right)=100\;\left(\%\right),$$

where *E*(λ), *T*(λ), and *R*(λ) are the extinction, transmittance, and reflectance, respectively, at a given wavelength.

## 3. Results and Discussion

### 3.1. Material Characterization of ZrO_2_ and ZrO_2_@SiO_2_ NPs

**Figure 2:**
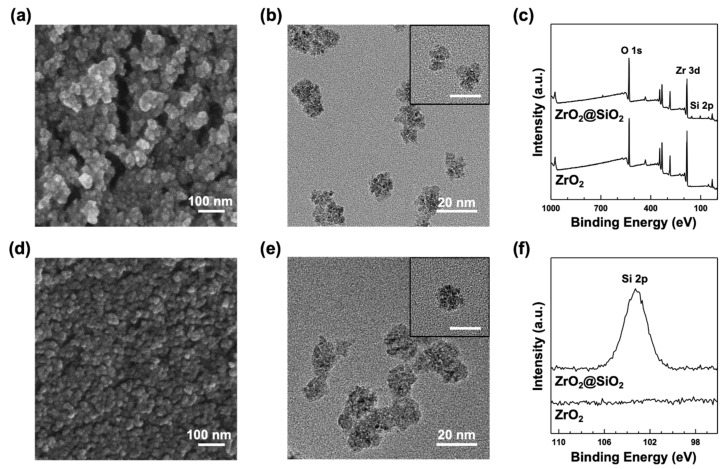
(**a**) SEM and (**b**) TEM images of synthesized ZrO_2_ NPs. The inset shows a representative TEM image of an individual ZrO_2_ NP with a 20 nm scale. (**d**) SEM and (**e**) TEM images of ZrO_2_@SiO_2_ NPs. The inset shows a representative TEM image of an individual ZrO_2_@SiO_2_ NP with a 20 nm scale. (**c**) Low-resolution XPS spectra of ZrO_2_ and ZrO_2_@SiO_2_ NPs. (**f**) High-resolution XPS spectra of Si 2p for both ZrO_2_ and ZrO_2_@SiO_2_ NPs.

The crystal structure of synthesized ZrO_2_ NPs was identified as the tetragonal phase, as shown in Figure S1, with XRD analysis [37,38]. The morphologies of the ZrO_2_ and ZrO_2_@SiO_2_ NPs were investigated using SEM and TEM. As shown by SEM images (Figure 2a,d), the shapes of the ZrO_2_ NPs were relatively irregular compared to those of the ZrO_2_@SiO_2_ NPs. NPs were agglomerated and it was difficult to find individual units of NPs. In contrast, individual units of ZrO_2_@SiO_2_ NPs were easily found in the SEM image. From the TEM analysis, the diameters of ZrO_2_ and ZrO_2_@SiO_2_ NPs were measured to be 13.7 ± 3.1 nm (Figure 2b) and 16.3 ± 3.4 nm (Figure 2e), respectively. The SiO_2_ layer was approximately calculated from the radius difference of the ZrO_2_ NPs and ZrO_2_@SiO_2_ NPs as 1.3 ± 2.3 nm. In addition, TEM and EDX analysis showed that Zr and Si coexisted in ZrO_2_@SiO_2_ NPs (Figure 1b), indicating that the surface of ZrO_2_ NPs was modified with an nm-thick SiO_2_ layer with the Stöber method.

The elemental analysis of ZrO_2_ and ZrO_2_@SiO_2_ NPs was performed using XPS. The corresponding low- and high-resolution XPS spectra are shown in Figure 2c,f, respectively. ZrO_2_@SiO_2_ NPs exhibited peaks of Zr (10.2%), Si (5.5%), O (47.7%), and C (36.6%), with a Zr:Si atomic ratio of 1.9. Zr3d_5/2_ peaks in both NPs were detected at 182.6 and 182.7 eV, respectively, which were assigned to the binding energy of Zr^4+^. The peaks of O1s were detected at 530.6 and 531.9 eV, respectively, which were assigned to the binding energy of Zr–O [39]. No significant signal of Si2p was observed in the ZrO_2_ NPs; however, the ZrO_2_@SiO_2_ NPs showed the peak of Si2p at 103.3 eV, which was assigned to the binding energy of Si–O [39].

### 3.2. The Surface Modification of ZrO_2_ NPs with the SiO_2_ Layer Controlled by the Reaction Time

**Figure 3:**
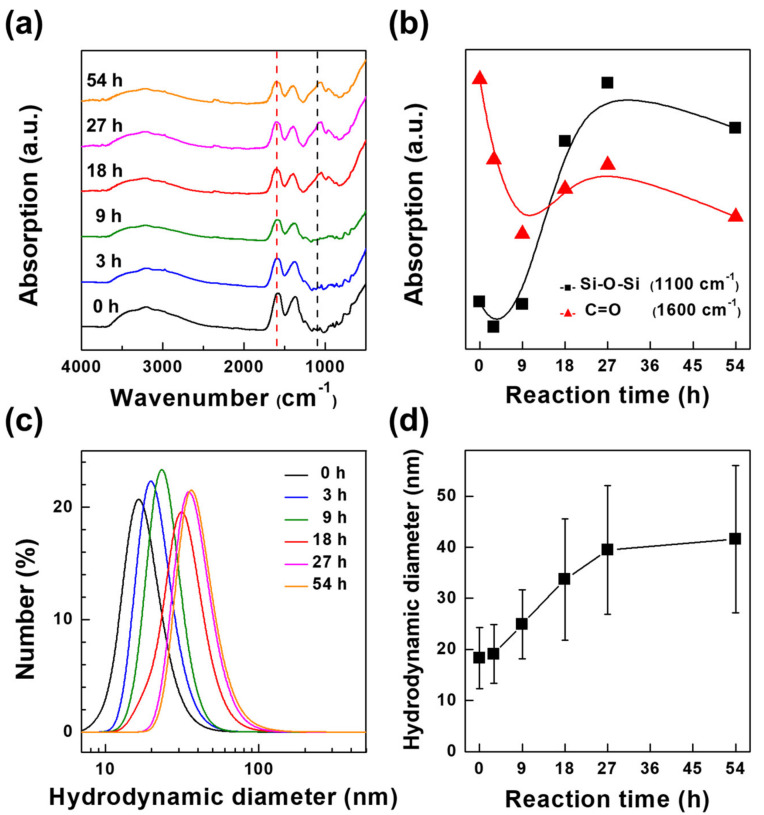
(**a**) FT-IR spectra of ZrO_2_@SiO_2_ NPs with different reaction times. Dashed lines indicate the peak positions corresponding to (black) Si–O–Si and (red) C=O bonds, respectively. (**b**) Changes in absorption intensities of ZrO_2_@SiO_2_ NPs according to the reaction time for (black) Si–O–Si and (red) C=O bonds. (**c**) Size distribution (hydrodynamic diameter) of ZrO_2_@SiO_2_ NPs with different reaction times, measured by DLS analysis. (**d**) Changes in mean hydrodynamic diameter of ZrO_2_@SiO_2_ NPs according to the reaction time. The error bar represents the standard deviation in the hydrodynamic diameters of the NPs.

As described above, the surface modification of ZrO_2_ NPs with the SiO_2_ layer was controlled by varying the reaction time of the Stöber method [22]. FT-IR and DLS measurements were conducted to monitor the increase in the size of the ZrO_2_@SiO_2_ NPs as a result of the surface modification of the ZrO_2_ NPs with the SiO_2_ layer with the reaction time. The FT-IR spectra of the ZrO_2_@SiO_2_ NPs at different reaction times are shown in Figure 3a. Peak intensities observed at 1600 cm^−1^ and 1100 cm^−1^ can be attributed to the C=O stretching from residual citric acid on the ZrO_2_ NP surface and the Si–O–Si asymmetric stretching from SiO_2_, respectively [40,41]. As the reaction time increased, the absorption in the 1600 cm^−1^ region decreased, while the absorption in the 1100 cm^−1^ region increased, supporting the hypothesis that the growth of the SiO_2_ layer can be controlled by the reaction time (Figure 3b). DLS spectra of ZrO_2_@SiO_2_ NPs show that the hydrodynamic size of the ZrO_2_@SiO_2_ NPs gradually increased with the reaction time up to 27 h, as shown in Figure 3c,d. The hydrodynamic sizes of the ZrO_2_ NPs and ZrO_2_@SiO_2_ NPs were larger than the physical sizes of the NPs determined from TEM analysis (Figure 2b,e). This was because the hydrodynamic size included not only the physical size of the NPs but also the thickness of the double layer, composed of the stern layer and the diffusion layer, on the NPs [42]. The hydrodynamic size was used limitedly to describe the NP size and DLS analysis showed that the surface modification of ZrO_2_ NPs with a SiO_2_ layer could be reproducibly controlled using the reaction time [42].

### 3.3. Optical Characterization of ZrO_2_ and ZrO_2_@SiO_2_ NPs

**Figure 4:**
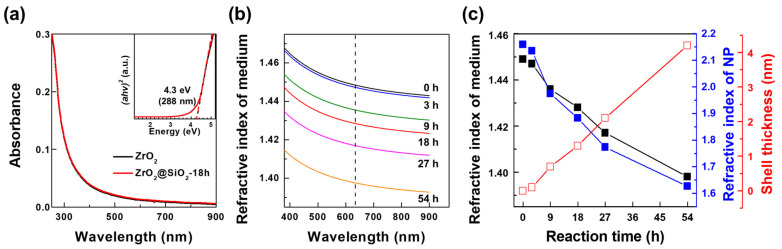
(**a**) UV-vis absorption spectra of (black) ZrO_2_ and (red) ZrO_2_@SiO_2_-18h NPs. The inset shows the corresponding Tauc plots for (black) ZrO_2_ and (red) ZrO_2_@SiO_2_-18h NPs. (**b**) Refractive index dispersions of aqueous solutions of ZrO_2_@SiO_2_ NPs prepared with different reaction times. All solutions were prepared with 50 wt% NP content. (**c**) Refractive (black) indices (at λ = 633 nm) for ZrO_2_@SiO_2_ NP solutions with different reaction times and (red) calculated SiO_2_ layer thicknesses, as well the (blue) effective refractive indices of ZrO_2_@SiO_2_ NPs.

The effects of the surface modification of ZrO_2_ NPs with different reaction times on optical properties, including the transmittance and effective refractive index, were evaluated using UV-vis spectroscopy and ellipsometry (Figure 4). UV-vis spectra of aqueous solutions of ZrO_2_ and ZrO_2_@SiO_2_-18h NPs were obtained in the wavelength range from 250 to 900 nm (Figure 4a). The spectral features of the ZrO_2_ and ZrO_2_@SiO_2_ NP aqueous solutions were almost identical; hence, light absorption by the SiO_2_ layer was negligible. In both spectra, light absorption increased rapidly at below 300 nm, and the bandgap energy of 4.3 eV (288 nm) was determined from the Tauc plot (Figure 4a (inset)) [43]. Thus, the light absorption of NPs can be ignored in the visible range [44].

To determine the refractive index dispersions of the NP aqueous solutions, psi and delta values were measured with the ellipsometer and fitted using the Cauchy model. The Cauchy model is appropriate for describing the refractive index dispersion in the wavelength region where the light absorption is negligible, as shown in the following equation:

(2) $$n\left(\lambda \right)=A+\frac{B}{\lambda^{2}}+\frac{C}{\lambda^{4}},$$

where A, B, and C are constants; λ is the wavelength; and *n*(λ) is the refractive index at a given wavelength [45]. The refractive index dispersions of the 50 wt% ZrO_2_@SiO_2_ NP aqueous solutions for each reaction time are shown in the wavelength range from 380 to 900 nm in Figure 4b. The refractive indices of each NP solution after 0, 3, 9, 18, 27, and 54 h were determined as 1.45, 1.45, 1.44, 1.43, 1.42, and 1.40 at λ = 633 nm, respectively (the black line in Figure 4c, Table 1) [34]. Among the various effective refractive index approximations, the effective medium approximation (Equation (3)) gave calculated values for the effective refractive indices of the NP solutions closest to the experimental values measured in the ellipsometry analysis (Figure S2) [34,46,47,48,49,50,51,52]. The effective medium approximation (Equation (3)) was used as a simple model to calculate the effective refractive indices of ZrO_2_@SiO_2_ NPs for each reaction time (the blue line in Figure 4c), considering the refractive index of bare ZrO_2_ NPs and a matrix (water) and the volume fraction of NPs in the solution (Figure S2, Table S1) [34,46,47,48,49]:

(3) $$n_{eff}\left(\lambda \right)=\varphi n_{NP}\left(\lambda \right)+\left(1-\varphi \right)n_{matrix}\left(\lambda \right),$$

where *n_NP_*(λ), *n_matrix_*(λ), and *n_eff_*(λ) are the refractive indices of the NPs, matrix, and NP solution, respectively, at a given wavelength. φ is the volume fraction of NPs in the NP solution. φ values were calculated as 0.141, 0.144, 0.161, 0.175, 0.192, and 0.244 for reaction times of 0, 3, 9, 18, 27, and 54 h, respectively. The refractive indices of each of ZrO_2_@SiO_2_ NP solution for the reaction times of 0, 3, 9, 18, 27, and 54 h were calculated as 2.16, 2.14, 1.98, 1.88, 1.77, and 1.63, respectively [34]. The refractive indices of the ZrO_2_@SiO_2_ NPs decreased with the reaction time and the surface modification of the ZrO_2_ NPs. This was because the volume fraction of the high-refractive-index ZrO_2_ (*n* = 2.16) decreased relatively and that of the low-refractive-index SiO_2_ (*n* = 1.46) increased with the increasing reaction time during the surface modification of NPs, considering the same NP weight content in aqueous solutions. The shell thicknesses of the SiO_2_ layers of the ZrO_2_@SiO_2_ NPs for each reaction time of 0, 3, 9, 18, 27, and 54 h were calculated as 0.0, 0.1, 0.7, 1.3, 2.1, and 4.2 nm, respectively (the red line in Figure 4c) [34,46,47,48,49]. In particular, the calculated shell thickness of the ZrO_2_@SiO_2_ NPs for the reaction time of 18 h (ZrO_2_@SiO_2_-18h) was consistent with that given by the TEM analysis, as described above (Figure 2b,e).

### 3.4. Dispersibility of ZrO_2_@SiO_2_ NPs in PDMS

Since PDMS is a hydrophobic polymer matrix with a high molecular weight, surface modification is required for ZrO_2_ NPs, which have COO^−^ groups on the surface, to prevent the agglomeration of NPs in the PDMS matrix. ZrO_2_@SiO_2_ NPs can form chemical affinity with PDMS through the formation of bonds between the surficial Si–OH groups of NPs and PDMS chains [53,54,55]. Dispersion of ZrO_2_@SiO_2_ NPs in the PDMS matrix can be improved as the surfaces of the NPs are subjected to greater modification by the SiO_2_ layer. However, to maintain a high refractive index, the SiO_2_ layer for the ZrO_2_@SiO_2_ NPs should preferably be thin; therefore, determining the appropriate thickness of the SiO_2_ layer is essential to achieve both transparency and a high refractive index in the nanocomposite film.

**Figure 5:**
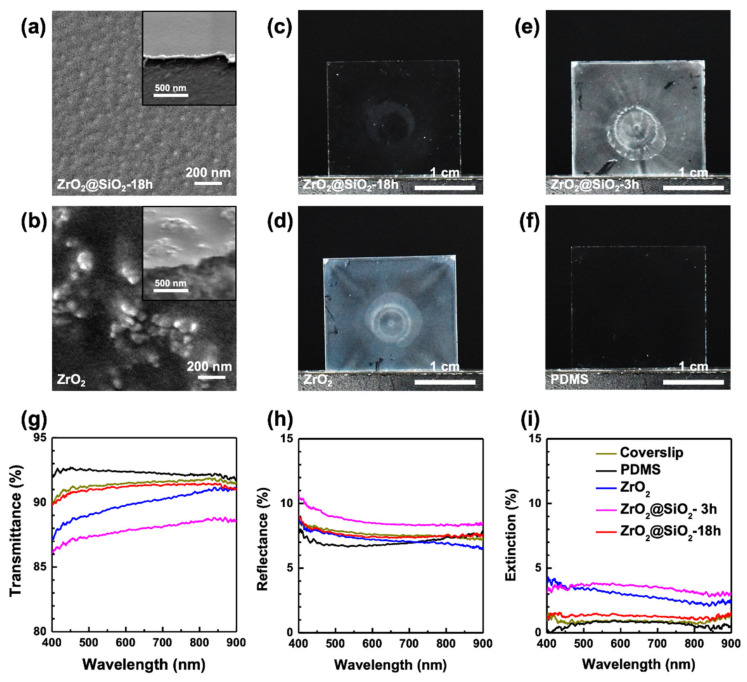
SEM images of (**a**) ZrO_2_@SiO_2_-18h-PDMS and (**b**) ZrO_2_-PDMS films. The inset shows 70 degree SEM images of the film edges of (**a**) ZrO_2_@SiO_2_-18h-PDMS and (**b**) ZrO_2_-PDMS films. Digital photos of (**c**) ZrO_2_@SiO_2_-18h-PDMS, (**d**) ZrO_2_-PDMS, (**e**) ZrO_2_@SiO_2_-3h-PDMS, and (**f**) PDMS films. All films were prepared with 100 nm thickness on Si substrates or coverslips with NP contents of 50 wt%. (**g**) Transmittance (T), (**h**) reflectance (R), and (**i**) extinction (E) spectra of corresponding films.

ZrO_2_@SiO_2_-PDMS nanocomposite films were prepared by dispersing ZrO_2_@SiO_2_ NPs subjected to different reaction times in the PDMS matrix and curing the mixture on a glass coverslip, as described above. SEM images and digital photographs were compared for the nanocomposite films to identify the dispersion of ZrO_2_@SiO_2_ NPs according to the surface modification (Figure 5). Figure 5a,b show top-view and (inset) 70 degree SEM images of 50 wt% ZrO_2_-PDMS and ZrO_2_@SiO_2_-18h-PDMS, respectively. It was found that the surface modification of NPs using SiO_2_ inhibited the agglomeration of the ZrO_2_ NPs in PDMS and induced a uniform dispersion. In the case of ZrO_2_@SiO_2_-18h-PDMS, ZrO_2_@SiO_2_-18h NPs were dispersed without any recognizable agglomeration. However, in the case of ZrO_2_-PDMS, ZrO_2_ NPs were heavily agglomerated into several hundred nanometers. The change in transparency of the nanocomposite films according to the surface modification of the ZrO_2_ NPs is shown in digital photographs in Figure 5c–e. ZrO_2_-PDMS appeared opaque due to light scattering from the agglomeration of NPs (Figure 5d). ZrO_2_@SiO_2_-3h-PDMS also appeared opaque, indicating that the surface modification of the NPs was insufficient to achieve good dispersion in PDMS (Figure 5e). On the other hand, ZrO_2_@SiO_2_-18h-PDMS was transparent, to a similar degree as the PDMS, showing that NPs were well-dispersed in the matrix (Figure 5c,f); that is, unlike the ZrO_2_ and ZrO_2_@SiO_2_-3h NPs, the ZrO_2_@SiO_2_-18h NPs had a sufficiently thick SiO_2_ layer, leading to homogenous dispersion in PDMS.

### 3.5. Optical Characterization of ZrO_2_@SiO_2_-PDMS

The optical properties of the nanocomposite films were characterized using the lab-built UV-vis spectrometer and the ellipsometer. The transmittance and reflectance spectra of the nanocomposite films were measured using films prepared on coverslips. The air was set to have a transmittance of 100%. The extinction spectra of the film were obtained as described in Equation (1). The transmittance, reflectance, and extinction spectra of 50 wt% ZrO_2_@SiO_2_-PDMS are shown in Figure 5g–i, respectively. Transmittances of the coverslip, PDMS, and ZrO_2_@SiO_2_-18h-PDMS (50 wt%) were, averaged in the wavelength region from 400 to 800 nm, 91.4%, 92.3%, and 91.1%, respectively (Figure 5g). These results were consistent with the digital photographs shown in Figure 5c,f. The reflectance value for the coverslip, PDMS, and ZrO_2_@SiO_2_-18h-PDMS were 7.7%, 7.1%, and 7.6%, respectively (Figure 5h). Extinction spectra were obtained based on the transmittance and reflectance to compare the opaqueness of the nanocomposite films. The extinction values of the coverslip, PDMS, and ZrO_2_@SiO_2_-18h-PDMS were 0.9%, 0.7%, and 1.3%, respectively (Figure 5i). In contrast, 50 wt% ZrO_2_-PDMS and ZrO_2_@SiO_2_-3h-PDMS exhibited higher extinction than 50 wt% ZrO_2_@SiO_2_-18h-PDMS owing to the large agglomerations of NPs.

**Figure 6:**
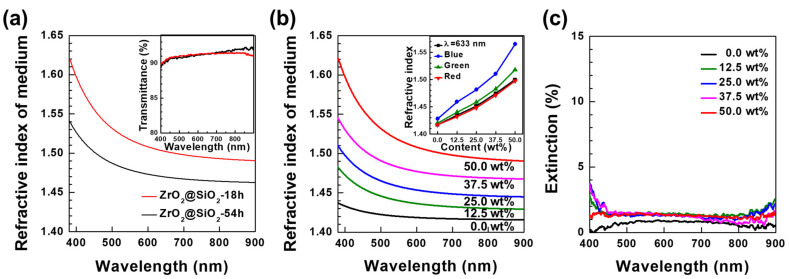
(**a**) Refractive index dispersions of (red) ZrO_2_@SiO_2_-18h-PDMS and (black) ZrO_2_@SiO_2_-54h-PDMS nanocomposite films. Films were prepared with particle contents of 50 wt%. The inset shows the transmittance spectra of the corresponding nanocomposite films. (**b**) Refractive index dispersions of ZrO_2_@SiO_2_-18h-PDMS films with different NP contents (0.0 to 50.0 wt%). The inset shows the refractive indices (at (black) λ = 633 nm and RGB wavelengths selected according to the CIE 1931 color space (blue—λ = 436 nm, green—λ = 546 nm, and red—λ = 700 nm) of the corresponding nanocomposite films. (**c**) Extinction spectra of the corresponding nanocomposite films.

Ellipsometry was performed to obtain the refractive index dispersion of the prepared nanocomposite films, as shown in Figure 6 and Figure S3. The refractive index of 50 wt% ZrO_2_-PDMS could not be obtained because of strong light scattering from the agglomeration of NPs. In the case of 50 wt% ZrO_2_@SiO_2_-3h-PDMS, the refractive index could only be measured in some locations. On the other hand, the refractive indices of both 50 wt% ZrO_2_@SiO_2_-18h-PDMS and ZrO_2_@SiO_2_-54h-PDMS could be measured in every location because NPs were well-dispersed without any hindrance from light scattering, as shown in Figure 6a. The difference between the refractive indices of ZrO_2_@SiO_2_-18h-PDMS and ZrO_2_@SiO_2_-54h-PDMS was 0.3 at 633 nm, which is consistent with the result shown in Figure 4c. The average transmittances of 50 wt% ZrO_2_@SiO_2_-18h-PDMS and ZrO_2_@SiO_2_-54h-PDMS were 91.1% and 91.3%, respectively; i.e., they had almost the same transmittance (Figure 6a (inset)). The Stöber method with the reaction time of 18 h was selected as optimal for the surface modification of ZrO_2_ NPs in this work because ZrO_2_@SiO_2_-18h-PDMS had excellent transmittance and high refractive indices.

Finally, the refractive index dispersions for ZrO_2_@SiO_2_-18h-PDMS were measured at different NP contents of 0.0, 12.5, 25.0, 37.5, and 50.0 wt%, as shown in Figure 6b. The refractive indices of ZrO_2_@SiO_2_-18h-PDMS with different NP contents were represented at a wavelength of 633 nm and at RGB colors selected according to the CIE 1931 color space; red—λ = 700 nm, green—λ = 546 nm, and blue—λ = 436 nm (Figure 6b (inset), Table S2) [56]. By changing the ZrO_2_@SiO_2_-18h NP content, the refractive index of the ZrO_2_@SiO_2_-18h-PDMS was adjusted from 1.42 to 1.60 in the visible-light region. The light extinction of ZrO_2_@SiO_2_-18h-PDMS was 1.5% or lower, indicating that ZrO_2_@SiO_2_-18h-PDMS achieved excellent transparency with all the studied NP contents (Figure 6c). Scratch resistance is one of the mechanical properties that the developed nanocomposite must obtain in order to be widely used as an advanced optical material. As shown by the scratch test depicted in Figure S5, the ZrO_2_@SiO_2_-18h-PDMS film seemed to have resistance similar to that of a bare PDMS film. It is necessary to improve the surface resistance and mechanical properties of the thin nanocomposite film through follow-up studies on the dispersion of ZrO_2_@SiO_2_ NPs in polymer matrixes that are harder than PDMS, including PET, epoxy, and acrylic resins.

## 4. Conclusions

We demonstrated that ZrO_2_ NPs could be stably and homogeneously dispersed in PDMS only after surface modification with a thin SiO_2_ layer using the simple Stöber method. We fabricated ZrO_2_@SiO_2_-PDMS nanocomposite films with adjustable refractive indices and excellent transparency. ZrO_2_ NPs were synthesized via hydrothermal synthesis, and their surface modification with a nanometer-thick SiO_2_ layer was effectively controlled by adjusting the reaction time of the Stöber method. By dispersing surface-modified ZrO_2_@SiO_2_-18h NPs in the PDMS, thin nanocomposite films with high refractive indices and excellent transparency were obtained. The ZrO_2_@SiO_2_-18h-PDMS nanocomposite films exhibited excellent transparency (91.1%), close to that of the coverslip (91.4%) in the visible region, and adjustable refractive indices (1.42–1.60) for the ZrO_2_@SiO_2_ NP content. ZrO_2_@SiO_2_-PDMS nanocomposite films may be useful in developing advanced optical devices based on simple synthesis and fabrication methods.

## Figures and Tables

**Table 1:** Refractive indices measured from ZrO_2_@SiO_2_ NP solutions with different reaction times and effective refractive indices, densities, and SiO_2_ layer thicknesses calculated for ZrO_2_@SiO_2_ NPs. All refractive indices are represented at the wavelength of λ = 633 nm (see Supplementary Materials for details).

Reaction Time (h)	ZrO_2_@SiO_2_ NP Solution	ZrO_2_@SiO_2_ NP		
	Refractive Index	Refractive Index ^a^	Density ^a^ (g/mL)	SiO_2_ Layer Thickness ^a^ (nm)
0	1.45	2.16	6.10	0.0
3	1.45	2.14	5.98	0.1
9	1.44	1.98	5.19	0.7
18	1.43	1.88	4.74	1.3
27	1.42	1.77	4.20	2.1
54	1.40	1.63	3.47	4.2

^a^ Values were calculated using the diameter of ZrO_2_ NPs as determined by the TEM analysis (Figure 2) and the parameters given in Table S1.

## Data Availability

Data presented in this article are available upon request from the corresponding author.

## Supplementary Materials

The following supporting information can be downloaded from https://www.mdpi.com/article/10.3390/nano12142328/s1. Figure S1: XRD patterns of ZrO_2_ NPs; Figure S2: Refractive indices of ZrO_2_ NP aqueous solutions at λ = 633 nm with different reaction times; Table S1: Values from the literature for the refractive indices and density of ZrO_2_, SiO_2_, and water; Figure S3: Ellipsometric spectra of nanocomposite films; Table S2: Refractive indices of ZrO_2_@SiO_2_-18h-PDMS nanocomposite films prepared with different NP contents; Figure S4: Thicknesses of prepared nanocomposite and PDMS films; Figure S5: The scratch resistance tests for the ZrO_2_@SiO_2_-18h-PDMS nanocomposite film.
