# Supplementary material for: Surface Modification of ZrO2 Nanoparticles with TEOS to Prepare Transparent ZrO2@SiO2-PDMS Nanocomposite Films with Adjustable Refractive Indices
Source: Nanomaterials (Basel). 2022 Jul 6;12(14):2328. doi: 10.3390/nano12142328 (PMC9320452; doi:10.3390/nano12142328)
Supplement: Supplementary file 1 [file nanomaterials-12-02328-s001.zip › nanomaterials-1797755-supplementary.pdf]

Supplementary Materials

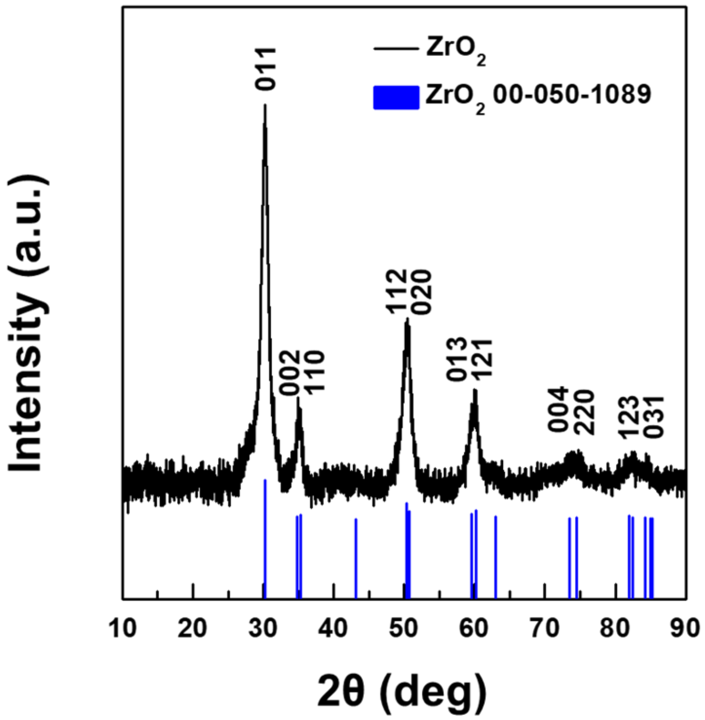

Figure S1. X-ray diffraction (XRD) pattern of ZrO_2. (black) Synthesized ZrO_2 NPs. (blue) Theoretical XRD pattern of tetragonal ZrO_2, PDF 00-050-1089 [37,38].

Calculation of the refractive index, density, and SiO_2 layer thickness of ZrO_2 NP and ZrO_2@SiO_2 NPs

When the shape of ZrO_2 and ZrO_2@SiO_2 NPs were assumed to be sphere, the volume fraction of ZrO_2 NPs in ZrO_2@SiO_2 NPs can be described by Equation S1

$$\varphi_{ZrO_2} = \frac{13.7^3}{(13.7+2x)^3} \quad , \tag{S1}$$

where the averaged diameter of ZrO_2 NPs is 13.7 nm and x is the SiO_2 layer thickness in nm and was initially set as 0.

The refractive index of ZrO_2@SiO_2 NPs can be calculated using **Equation 3** and the density of ZrO_2@SiO_2 NPs, $d_{ZrO_2@SiO_2}$, were calculated using **Equation S2**

$$d_{ZrO_2@SiO_2} = d_{ZrO_2} \times \varphi_{ZrO_2} + d_{SiO_2} \times (1-\varphi_{ZrO_2}) \quad . \tag{S2}$$

Then, the volume fraction of ZrO_2@SiO_2 NPs in 50 wt% aqueous NP solutions were calculated using **Equation S3.**

$$\varphi_{ZrO_2@SiO_2} = \frac{d_{water}}{d_{ZrO_2@SiO_2} + d_{water}} \quad . \tag{S3}$$

The effective refractive indices of 50 wt% NP solutions were calculated using **Equation S3** with refractive indices of ZrO_2@SiO_2 NPs and water. All the calculations were repeated until the difference between calculated and experimental effective refractive indices of 50 wt% NP solutions were minimized with different SiO_2 layer thickness in the range from 0 to 5.0 nm. All of the refractive index, density, and SiO_2 layer thickness of ZrO_2@SiO_2 NPs of these minimized difference were summarized in Table 1.

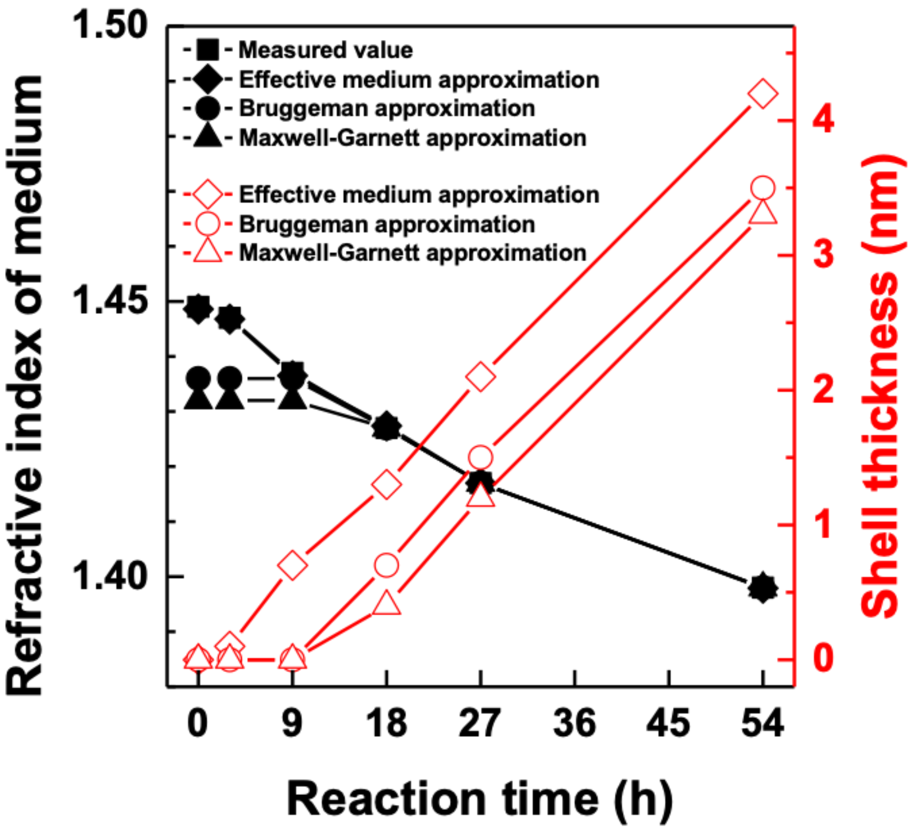

Figure S2. Measured and calculated effective refractive index and shell thickness of 50 wt% aqueous solution of ZrO_2@SiO_2 NP with different reaction times. (black) effective refractive index and (red) shell thickness was calculated corresponding approximation model such as effective medium approximation, Bruggeman approximation, and Maxwell-Garnett approximation [34,50-52].

Table S1. Literature values of refractive index and density for ZrO_2, SiO_2, and water. These values were used to calculate the refractive index, density, and SiO_2 layer thickness of ZrO_2@SiO_2 NPs as shown in Figure 4c and Table 1.

Material	Refractive index (λ=633 nm)	Density (g/mL)	Ref
ZrO_2	2.16	6.10	[46,47]
SiO_2	1.46	2.65	[48]
Water	1.33	1.00	[49]

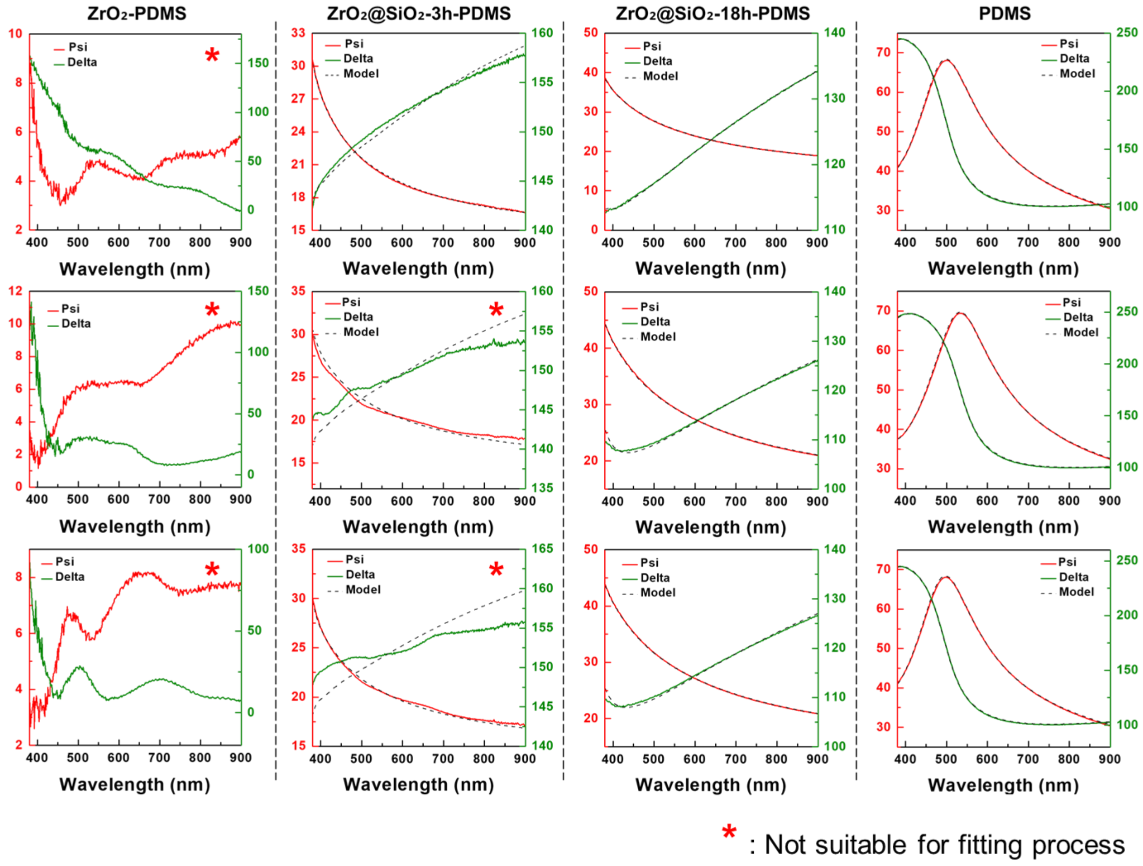

Figure S3. Ellipsometric spectra of (red) psi and (green) delta of nanocomposite and PDMS films, measured on three different sample locations. Psi and delta values were fitted with (dashed-black) Cauchy dispersion model. All nanocomposite films were prepared with NP content of 50 wt%.

Table S2. Refractive indices of ZrO_2@SiO_2-18h-PDMS nanocomposite films prepared with different NP contents. Refractive indices were determined at wavelengths of λ = 633, 436, 546, and 700 nm. The wavelength of (blue) 436, (green) 546, and (red) 700 nm were selected following CIE 1931 color system [56].

**ZrO_2@SiO_2-18h NP content (wt%)**	**Refractive index**			
	**λ=633 nm**	**λ=436 nm (blue)**	**λ=546 nm (green)**	**λ=700 nm (red)**
0.0	1.42	1.43	1.42	1.42
12.5	1.43	1.46	1.44	1.43
25.0	1.45	1.48	1.46	1.45
37.5	1.47	1.51	1.48	1.47
50.0	1.50	1.57	1.52	1.50

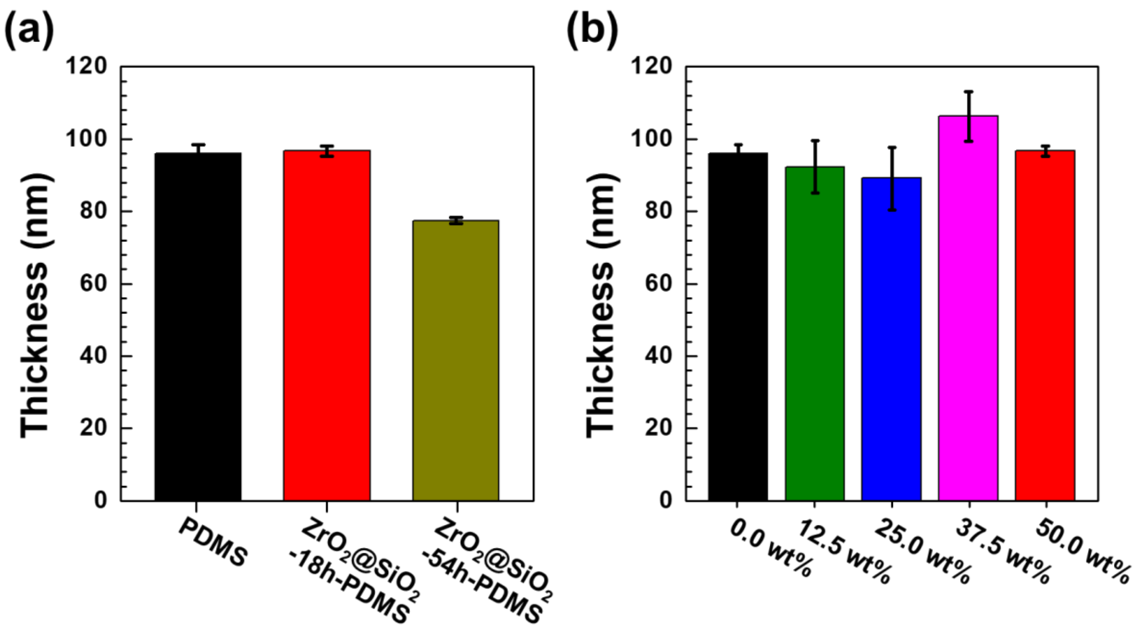

Figure S4. Thickness of prepared nanocomposite and PDMS films. (a) Film thickness of PDMS, ZrO_2@SiO_2-18h-PDMS (50 wt%), and ZrO_2@SiO_2-54h-PDMS (50 wt%). (b) Film thickness of ZrO_2@SiO_2-18h-PDMS according to NP contents (0.0–50 wt%). The error bar represents the standard deviation of film thickness.

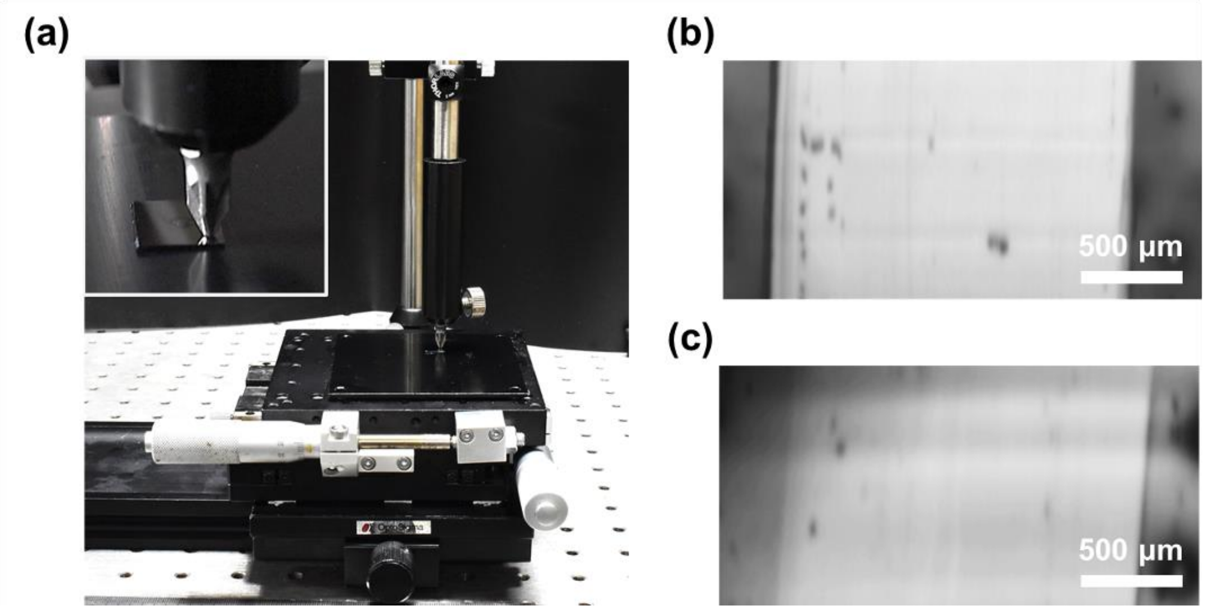

Figure S5. (a) Digital photograph of DIY scratch test setup. Optical microscope images (20× magnification) of (b) a bare PDMS film and (c) a 50 wt% ZrO_2@SiO_2-18h-PDMS films taken after the scratch test. Totally, the weight of 160 g was load on PDMS and nanocomposite films via a metal screwdriver bit.
